# Editorial: Behaviors and Neural Circuits in Sleep and Sedation

**DOI:** 10.3389/fnins.2022.930591

**Published:** 2022-06-03

**Authors:** Edward C. Harding, Zhe Zhang, Hailong Dong, Xiao Yu

**Affiliations:** ^1^Wellcome-MRC Institute of Metabolic Science, University of Cambridge, Cambridge, United Kingdom; ^2^Department of Life Sciences, Imperial College London, London, United Kingdom; ^3^Institute of Neuroscience, State Key Laboratory of Neuroscience, Center for Excellence in Brain Science and Intelligence Technology, Chinese Academy of Sciences, Shanghai, China; ^4^Department of Anesthesiology and Perioperative Medicine, Xijing Hospital, Fourth Military Medical University, Xi'an, China; ^5^Department of Basic and Clinical Neuroscience, Maurice Wohl Clinical Neuroscience Institute, Institute of Psychiatry, Psychology and Neuroscience, UK Dementia Research Institute, King's College London, London, United Kingdom

**Keywords:** sleep, thermoregulation, general anesthesia, neuronal circuitry, preoptic hypothalamus, sleep function, non REM (NREM) sleep, rapid eye movement (REM)

## Introduction

The function of sleep is an enduring mystery. Research over the last few years has attempted to unravel its complexity in the mammalian brain and determine the molecular and neuronal underpinnings of sleep state transitions.

This topic puts together new research that improves our understanding of the neural circuitry of sleep and its associated behaviors. Our long-term view is that understanding circuitry will drive our understanding of why we spend one third of our lives unconscious and vulnerable; unable to perform crucial biological functions from eating to hunting, foraging and reproduction. We seek to understand why such a state could be so vital as to be observed in all complex life.

## Contributions of Articles in This Series

The gating of sleep requires the integration of permissive signals from the environment, such as satiety status and ambient temperature, but how these are assimilated is unclear (Harding et al., 2019). Guillaumin and Burdakov have considered the role of neuromodulation in slow local microcircuits, across the hypothalamus, with a particular focus on peptide neuromodulators. As these peptides have a persistent extracellular presence, they are uniquely placed to facilitate the integration of these context-dependent permissive signals. This is an appealing hypothesis as a distinct feature of the hypothalamus is the diversity and abundance of neurons using neuropeptides in transmission (de Lecea et al., 1998; Svensson et al., 2019). Understanding the functions of these peptides may help us explain, for example, why galanin expressing neurons in the preoptic area appear to have a role in both stereotyped parental behavior, such as pup grooming, as well as the induction of non-rapid eye movement sleep (NREM) (Kohl et al., 2018; Kroeger et al., 2018; Ma et al., 2019; Reichert et al., 2019).

Disruption of neuropeptide transmission is most well understood in the case of the peptide orexin and the loss of orexin producing neurons *in vivo* results in narcolepsy. This peptide can also induce action potentials in the absence of neurotransmitter co-release, suggesting functions beyond neuromodulation (Schöne et al., 2014; Mahoney et al., 2019). Coffey et al., specifically considered the role of sex and age on the severity of narcolepsy in a doxycycline-inducible model of mouse narcolepsy, revealing interesting interactions with sex. We think this underlies the importance of considering these variables further in sleep research. The authors also propose that children with narcolepsy may suffer greater loss of orexin neurons than adults thus explaining the presence of more severe symptoms.

One of the most familiar aspects of natural sleep is that of waking-up and experiencing a lingering drowsiness, or “sleep-inertia.” Luppi et al., considered how emergence from anesthesia, at a lower dose than required for induction (e.g., “neural-inertia”), has similarities to sleep-inertia. The authors have proposed a new model of orexin and noradrenergic circuitry to mediate this process and help explain why elderly and narcoleptic patients are more susceptible to neural inertia following anesthesia (Scammell, 2003; Kelz et al., 2008; Silva and Duffy, 2008). Similarly, Wang L et al. have shown that activation of basal forebrain cholinergic neurons blunts normal sensitivity to propofol and shortens recovery from anesthesia. These neurons also induce wake-like signals in the medial prefrontal cortex.

Within the hypothalamus, specific populations are now well associated to sleep control and the gating of environmental cues. Harding et al. considered the contribution of a specific subset of preoptic neurons, that express nitric oxide synthase, to normal sleep and thermoregulatory cycles. Many NOS1 neurons are NREM active and, when synaptic transmission is blocked, bi-directional changes in sleep-wake propensity occur across the light-cycle, alongside a shift to slightly warmer core temperatures. In agreement with previous work, these neurons appear to have a role in gating sleep in relation to thermoregulatory responses to ambient temperature (Harding et al., 2018). The role of nitric oxide (NO), however, remains unclear. NO is a gaseous and transient neuromodulator, with diffusion distances of up to a few tens of microns. As such, NO may influence glutamatergic transmission through cGMP mediated changes in excitability or indirectly via local vascular smooth muscle, supporting vasodilation (Förstermann and Sessa, 2011).

Reitz and Kelz, have asked us to carefully consider the “shared circuitry hypothesis”; the idea that sleep circuits are hijacked by the actions of general anesthetics. They detail the challenges of attributing anesthetic action to a single neuronal population, given the ability of some preoptic neurons to induce wakefulness, as well as our newfound understanding of exceptional cellular heterogeneity in the preoptic region (Moffitt et al., 2018; Vanini et al., 2020).

Cheng et al. have carried out extensive pharmacological assessment of the flip-flop hypothesis (Saper et al., 2010). Consistent with this model, they show that VLPO neurons can be driven to induce NREM by targeted injection of L-glutamate, while this action can be blocked by injecting bicuculline into the TMN. Conversely, L-glutamate injection into the TMN during lights-ON induced wakefulness that could be blocked by triprolidine injection in the VLPO.

Finally, we are beginning to understand how sleep-deprivation and sleep-rebound are perceived and encoded in the brain Wang H. et al. recorded an impairment of fine motor control alongside impaired functional connectivity, observed in fMRI, following sleep deprivation in healthy adult men In contrast to the human experience, Xu et al. found that sleep-deprivation at the circuit level in rats, by direct activation of the medial parabrachial nucleus (MPB), does not always result in sleep-rebound. This has parallels to the lesioning of ventral tegmental area (VTA) *Vgat*-Cre neurons in mice, that also do not exhibit rebound sleep following sleep deprivation, showing clearly that these intrinsic properties of sleep can be decoupled (Yu et al., 2021). Furthermore, if only certain wake-active populations can induce sleep-rebound, are the waking-behaviors linked to these neurons also more important for the function of sleep?

## Conclusions and Perspective

This series has emphasized that molecular heterogeneity is an on-going challenge for understanding sleep circuitry. Common mouse lines expressing Cre recombinase (e.g., *Vgat*-Cre, *Vglut2*-cre) facilitate access to smaller, but still highly diverse group of neurons, complicating our interpretation (Moffitt et al., 2018). We should also carefully consider the role of neuropeptides, as well as potential neuromodulators such as nitric oxide, that may not be functioning within the normal synaptic cascade. These non-canonical pathways may underly the integration of permissive conditions to sleep such as warmth-seeking and satiety (Goldstein et al., 2018; Harding et al., 2018; Komagata et al., 2019).

Finally, general anesthetics and sedatives share many features with sleep but not all anesthetics use the same circuitry. This allows for the exciting possibility that some compounds may induce more natural sleep than others and further development may move us closer to a true sleep-inducing agent (Franks and Wisden, 2021).

## Author Contributions

EH wrote the manuscript with assistance from XY. XY initiated this article collection. ZZ and HD provided feedback on the manuscript. All authors were topic editors for this article series.

## Conflict of Interest

The authors declare that the research was conducted in the absence of any commercial or financial relationships that could be construed as a potential conflict of interest.

## Publisher's Note

All claims expressed in this article are solely those of the authors and do not necessarily represent those of their affiliated organizations, or those of the publisher, the editors and the reviewers. Any product that may be evaluated in this article, or claim that may be made by its manufacturer, is not guaranteed or endorsed by the publisher.
